# Granulomatous diseases of the breast and axilla: radiological findings with pathological correlation

**DOI:** 10.1007/s13244-017-0587-9

**Published:** 2018-02-05

**Authors:** Jeffery E. Illman, Simone B. Terra, Allison J. Clapp, Katie N. Hunt, Robert T. Fazzio, Sejal S. Shah, Katrina N. Glazebrook

**Affiliations:** 1 Department of Radiology, Mayo Clinic, Rochester, MN USA; 2 Department of Laboratory Medicine and Pathology, Mayo Clinic, Rochester, MN USA; 3 Sanford Broadway Clinic, Fargo, ND USA

**Keywords:** Breast, Granulomatous mastitis, Mammography, MRI, Ultrasonography

## Abstract

**Objectives:**

This article reviews our experience and describes the literature findings of granulomatous diseases of the breast and axilla.

**Methods:**

After approval of the Institutional Review Board was obtained, the surgical pathological records from January 2000 to January 2017 were searched for the keyword *granulomatous*. Clinical, imaging and histology findings were reviewed by both a fellowship-trained radiologist and a breast-imaging consultant radiologist, reviewing 127 patients (age range, 32–86 years; 126 women and 1 man).

**Results:**

Most common causes of granulomatous lesions of the breast and axilla included silicone granulomas 33% (*n* = 42), fat necrosis 29% (*n* = 37) and suture granulomas 11% (*n* = 14). In 16% (*n* = 20), no cause could be found and clinical history was consistent with idiopathic granulomatous mastitis. Other granulomatous aetiologies included granulomatous infections, sarcoidosis and Sjögren’s syndrome. Causes of axillary granulomatous disease were similar to the breast; however, a case of cat-scratch disease was found that only involved the axillary lymph nodes. They can have a variable appearance on imaging and may mimic malignancy with irregular masses seen on mammography, ultrasound and magnetic resonance imaging. Fistulas to the skin and nipple retraction can suggest chronicity and a granulomatous aetiology. Combination of clinical history, laboratory and imaging findings can be diagnostic.

**Conclusions:**

Granulomatous processes of the breast are rare. The diagnosis can, however, be made if there is relevant history (prior trauma, silicone breast implants, lactation), laboratory (systemic or infectious processes) and imaging findings (fistula, nipple retraction). Recognising these entities is important for establishing pathological concordance after biopsy and for preventing unnecessary treatment.

**Teaching points:**

- *Breast granulomatous are rare but can mimic breast carcinoma on imaging*
- *Imaging with clinical and laboratory findings can correctly diagnosis specific granulomatous breast diseases*
- *Recognition of the imaging findings allows appropriate pathological concordance and treatment*

## Introduction

With Institutional Review Board approval, a search of the surgical pathology records of approximately 17,000 breast biopsies performed at our institution from 1 January 2000 to 1 January 2017 yielded 127 patients (age range, 32–86 years; 126 women and 1 men) with granulomatous disease of the breast, and their imaging results were available for review by both a fellowship-trained radiologist and breast-imaging consultant radiologist. The search found records of 42 silicone granulomas, 37 fat necrosis, 20 idiopathic granulomatous mastitis, 14 suture granulomas, 1 *Corynebacterium*, 1 *Mycobacterium fortuitum* infection, 2 sarcoidosis and 1 Sjögren’s syndrome. Additionally, nine cases of granulomatous lymphadenitis where found: six of which were secondary to granulomatous causes involving the breast (for example, silicone granuloma), one was idiopathic and one patient was diagnosed with cat-scratch disease.

**Fig. 1:**
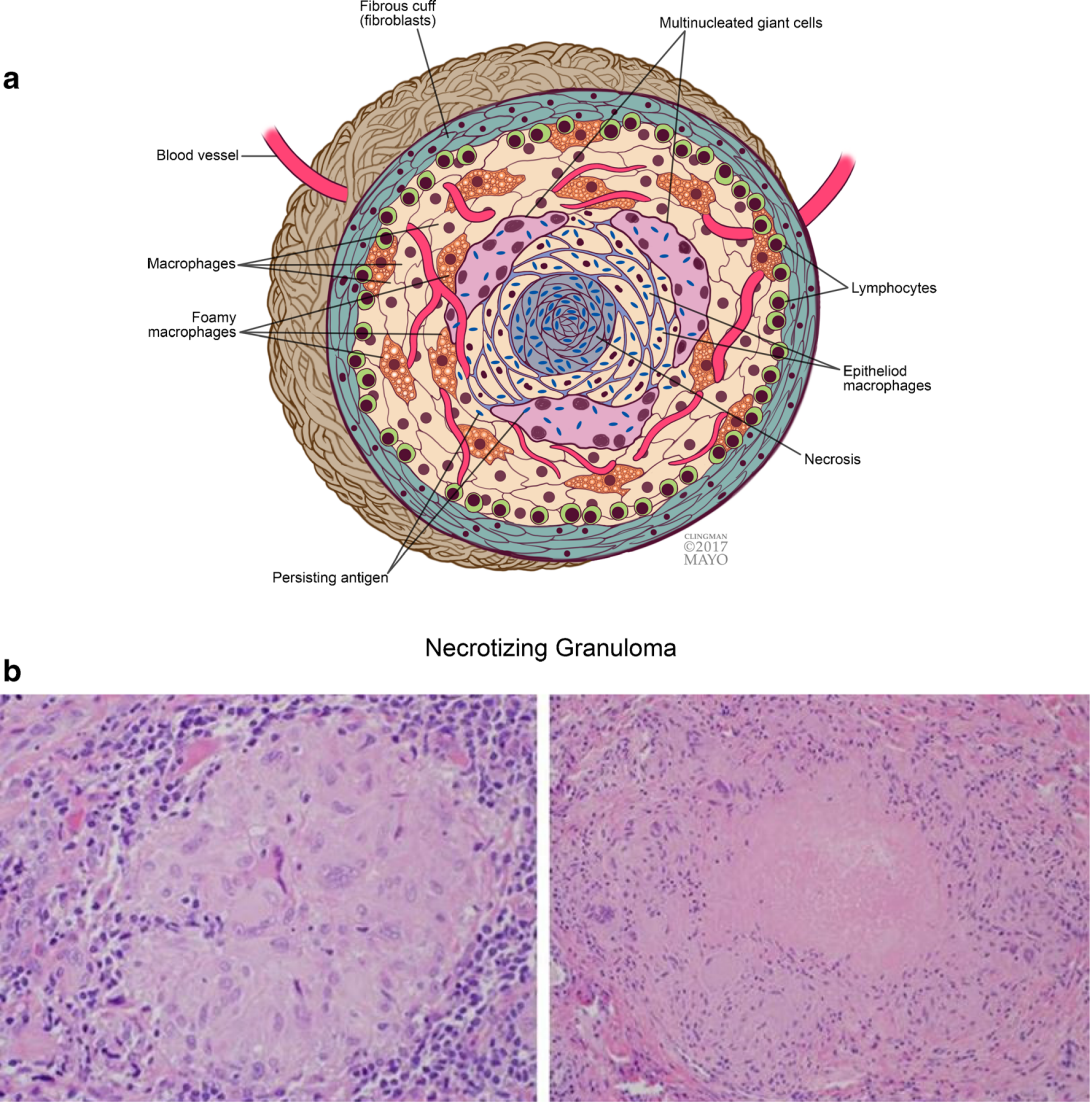
Granuloma. **a** Diagram of a granulomatous response (with central necrosis) to a persisting antigen. Persisting antigens include microorganisms, foreign material or antigens that are unknown at this time. (Reproduced with permission) **b **Histologic photomicrographs (original magnification x20, hematoxylin-eosin) of a nonnecrotizing granuloma (left) composed of lymphocytes, macrophages, and giant cells and a necrotizing granuloma (right) with central necrosis

A granuloma is the human immune system’s way of “walling off” an offending impurity, be it a foreign body, chronic infection or necrotic fat. A granuloma (Fig. 1) is an organised group of macrophages (mononuclear cells within tissue) associated with a variable amount of lymphocytes. The outer layer of the granuloma consists of lymphocytes, fibroblasts and vessels. Granulomas may also contain additional cells such as neutrophils, eosinophils and fibroblasts, which can provide a clue to the aetiology of the granuloma. Central necrosis may also help determine the cause of the granuloma; infectious granulomas tend to have central necrosis, termed *necrotising granulomas* (Fig. 1). Central necrosis with a cheese-like appearance has been termed *caseating necrosis* and is a feature of *Mycobacterium tuberculosis* infection. A non-caseating granuloma (Fig. 1) can occur with non-infectious causes such as sarcoidosis.

**Fig. 2:**
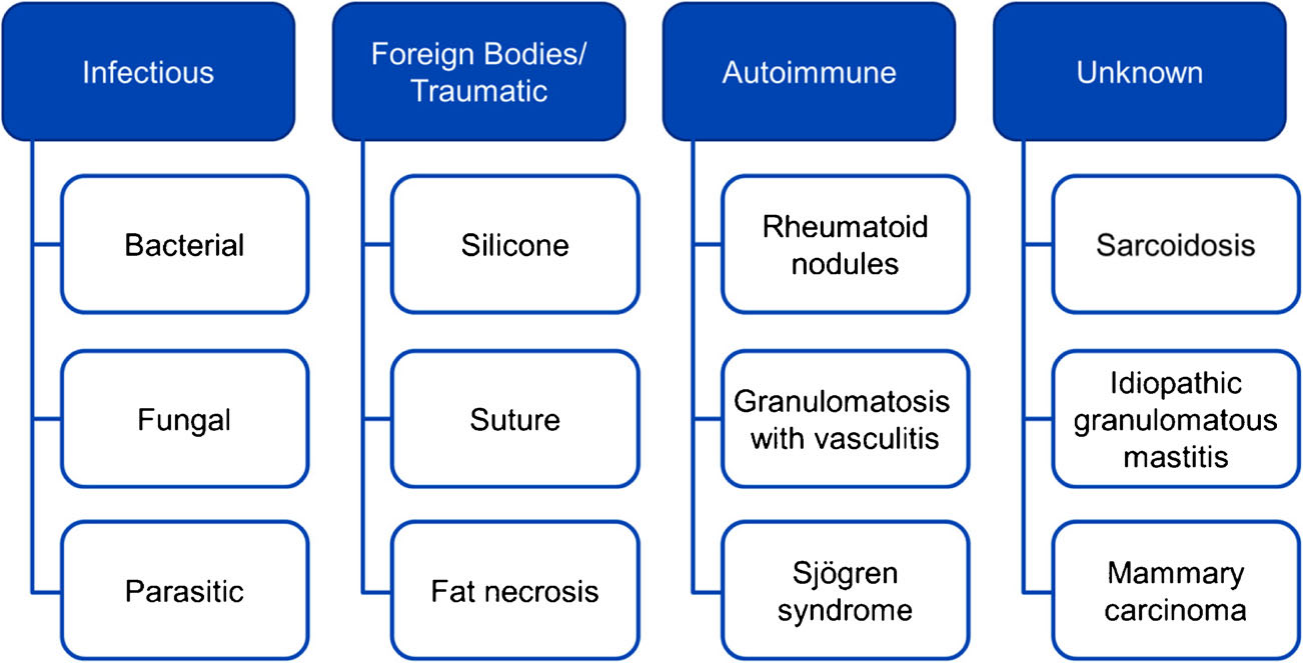
Categorisation. Causes of granulomatous inflammation within the breast and axilla

Imaging findings can have a variable appearance and may mimic malignancy with irregular masses on mammography, ultrasound and magnetic resonance imaging (MRI), requiring biopsy. The diagnosis can be suggested if correlation is made with clinical history. This article reviews and illustrates the salient imaging features of granulomatous processes of the breast and axilla, including infectious, traumatic, autoimmune, as well as unknown causes (Fig. 2).

## Infectious causes

Most breast infections are bacterial and are usually secondary to skin contamination, with most infections resolving without sequela. Specialised stains are occasionally necessary for specific diagnosis, including Gram, periodic acid-Schiff and Ziehl-Neelsen stains. Overall, chronic infections with granulomatous reactions are rare and a high index of clinical suspicion is necessary.

### Clinical

Clinically patients present with a painful and/or palpable breast mass, often with axillary lymphadenopathy. A discharging sinus tract can be seen if treatment is delayed. The subareolar regions may be relatively spared. Reactive lymphadenopathy may be present in up to 15% of cases.

### Mammography

Imaging features are variable depending on type of infection, but are unable to reliably separate based solely on imaging. Bacterial infections (*Corynebacterium*, etc.) often present as an erythematous, inflamed breast with skin thickening and complex cystic mass on ultrasound. An irregular hypoechoic mass or vague ill-defined hypoechoic mass with increased vascularity on Doppler evaluation can also be seen. Fungal infections (blastomycosis, cryptococcosis, histoplasmosis, actinomycosis) usually present as a lobulated, irregular or well-defined complex cystic mass sonographically. Less common parasitic infections (filarial, schistosomiasis, sparganosis, echinococcosis) can present with calcifications with or without a mass mammographically.

*Mycobacterium* infections (tuberculous, atypical) present with axillary lymphadenopathy, skin thickening or ill-defined mass, often with sinus tracts and fistulae. Mammographically, tuberculosis can manifest as an ill-defined or irregular mass resembling cancer; however, skin bulging and sinus tracts can suggest tuberculous infection and large, dense, calcified axillary lymph nodes can also be a clue to tuberculous infection.

### Ultrasound

**Fig. 3:**
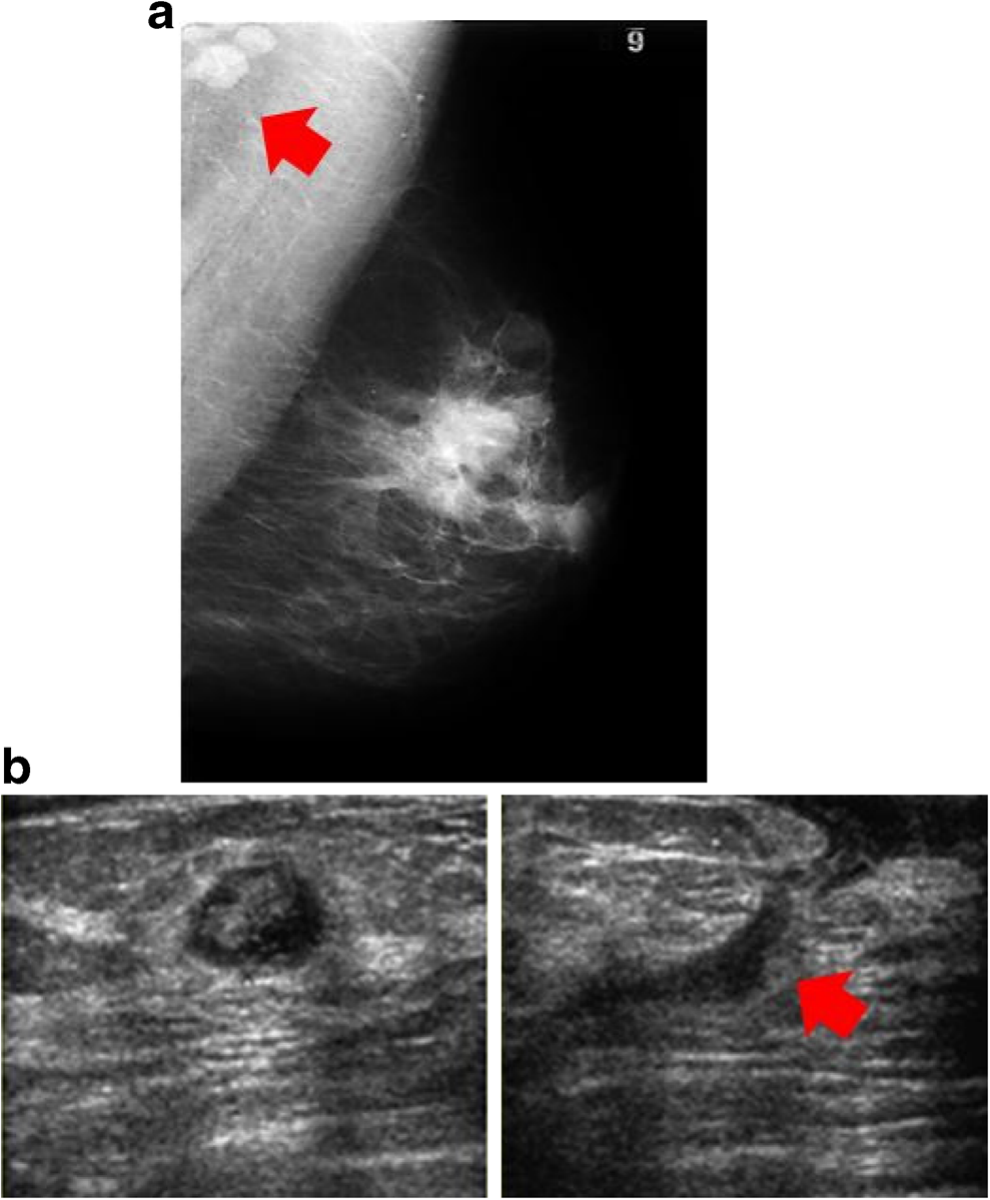
Infectious cause: *Mycobacterium fortuitum*. A 32-year-old woman had a new tender, palpable mass in the left breast. Biopsy showed granulomas, and purulent drainage from the needle tract was positive for *M. fortuitum*. Treatment with antibiotics, based on the sensitivity of the organism, led to resolution of the infection. **a** Left mediolateral-oblique view showed multiple masses with prominent left axillary lymph nodes (*red arrow*) and periareolar skin thickening. **b** Ultrasound showed a complex, hypoechoic fluid collection (*left*) with sinus tract extending to skin (*red arrow*) (*right*)

Ultrasonography is useful for evaluation of fistula or sinus tracts, and computed tomography (CT) or MRI is beneficial for assessing for chest wall involvement or invasion (Fig. 3). The coexistence of mammary tuberculosis and breast carcinoma has been reported [1, 2].

### MRI

On MRI, the most frequent finding was focal or diffuse asymmetrical signal intensity changes without significant mass effect. Nodular lesions can also be seen with hypointense T1 regions and hyperintense T2 regions. Dynamic contrast-enhanced MRI can show mass-like enhancement, ring-like enhancement and/or nodular enhancement, while time-intensity curves can vary.

### Histology

The granulomas can show caseating “cheesy” necrosis, and occasionally acid-fast bacilli are found within the central portion of the granuloma [1, 2]. Caseous necrosis is often encountered in tuberculosis infections but can also be caused by syphilis and certain fungi. A similar appearance can be associated with histoplasmosis, cryptococcosis and coccidioidomycosis.

## Foreign bodies and traumatic causes

Foreign body granuloma arises from reaction to any foreign materials that cannot be broken down, such as silicone, suture material, wood, gunpowder, plastic sewing needle and paraffin. Alternatively, traumatic granulomas originate from any procedure or injury resulting in fat necrosis. The granulomas that form around fat necrosis are the result of difficulty removing the necrotic fat because of its large size [3–6].

### Silicone

Silicone for breast augmentation was initially injected directly into the breast. This procedure was performed in the United States until it was prohibited in the 1970s because of reports of lymphadenopathy, infection, granulomatous masses and fibrosis.

Silicone implants were introduced in the 1960s with the theory that the barrier shell would reduce the complications of direct silicone injection. Unfortunately, the shells used in these early implants were semi-porous and allowed small amounts of silicone gel to “bleed” without implant rupture. These early implants were also prone to rupture with increasing age, and when they rupture result in large amounts of extracapsular silicone [7]. The complications of this free silicone were indistinguishable from those of direct silicone injection [8].

#### Clinical

Patients may present with pain, soreness or swelling in the affected breast, change in breast size or shape, lumps in the affected breast or softening or hardening of the affected breast. Silicone granulomas may present distant from the implant site; primarily, the ipsilateral chest wall and axillary lymph nodes are involved. Ductal extension, with silicone being discharged from the nipple, and pectoralis muscle extension have also been reported [9, 10].

#### Mammography

**Fig. 4:**
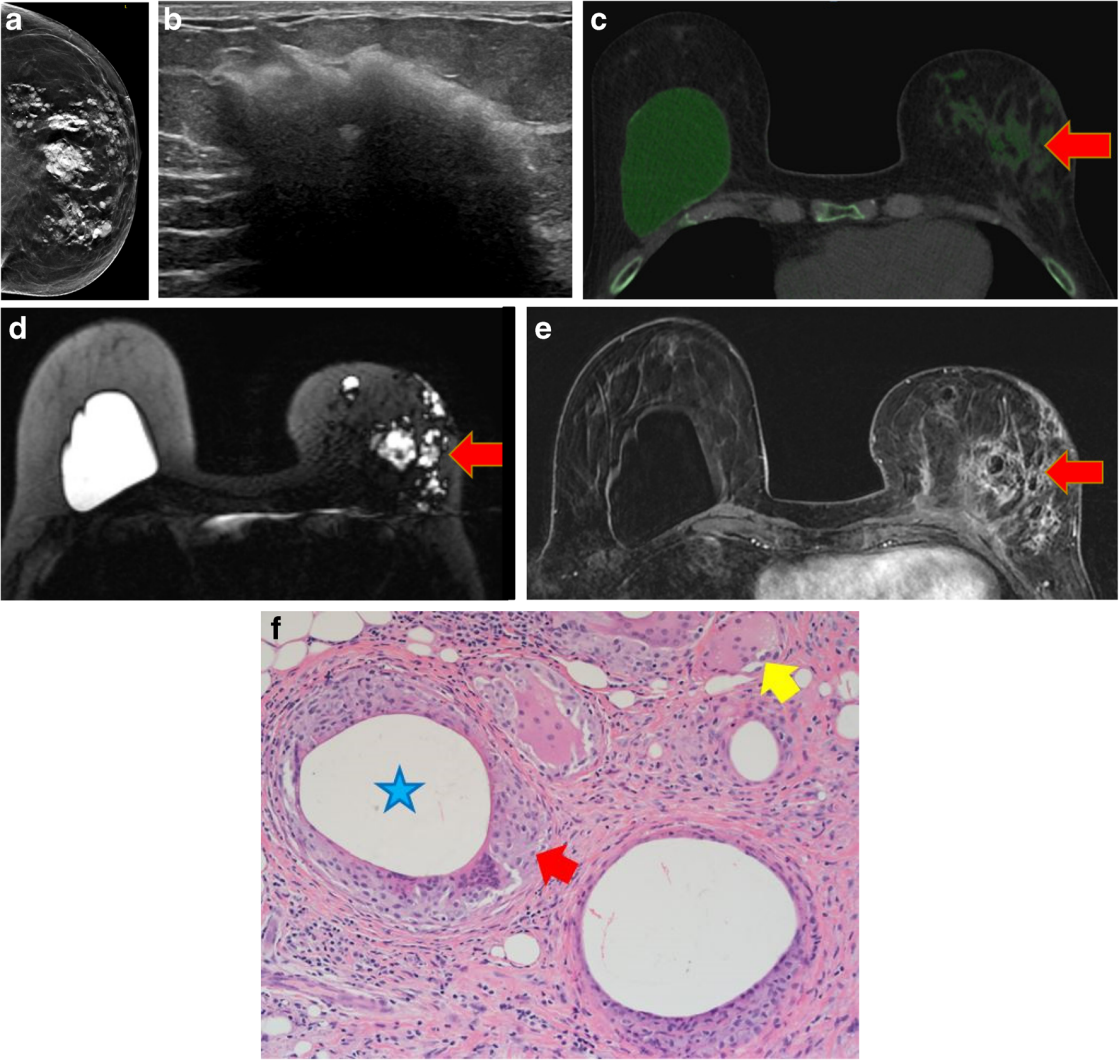
Silicone granuloma. A 60-year-old woman who has had bilateral breast subglandular silicone implantation 20 years ago, with removal of the left implant following extracapsular rupture 1 year previously. **a** Left craniocaudal view shows high density extracapsular silicone within the breast tissue. **b** Ultrasound of the inferior left breast shows echogenic mass with posterior shadowing—“snowstorm” appearance of free silicone. **c** Axial dual-energy CT with material decomposition and green pixilation to identify silicone, shows free silicone within the left breast (*red arrow*) with intact right implant. **d** Axial silicone-sensitive MR sequence demonstrates the same findings of extracapsular free silicone (*red arrow*) and intact right implant. **e** Axial post-gadolinium breast MRI showed non-enhancing extracapsular silicone with enhancement surrounding the silicone deposits, consistent with granulomatous inflammation (*red arrow*). **f** Histological photomicrograph (magnification ×20, haematoxylin-eosin) showed “siliconomas” with granulomas containing silicone particles (*blue star*), foreign body–type giant cells (*yellow arrow*), foamy macrophages and lymphocytes (*red arrow*)

On mammography, high-density masses are seen outside the implant margins (Fig. 4) and dense lymph nodes may be seen. Implant asymmetry or irregular contour are less specific signs, but can also be visualized.

#### Ultrasound

Ultrasonography shows a “snowstorm” appearance with well-defined echogenicity anteriorly and dirty posterior acoustic shadowing. The lymph nodes may also be echogenic with dirty shadowing.

#### CT

Dual-energy CT can perform material decomposition, identifying intracapsular and extracapsular rupture and extracapsular silicone [11].

#### MRI

MRI shows low T1-signal and high T2-signal foci separate from the implant and high-signal foci on silicone-sensitive sequences. Enhancement may be noted if active inflammation or granulomas are present.

### Fat necrosis

#### Clinical

Most cases of fat necrosis occur postoperatively or after radiation therapy. The condition is typically asymptomatic; however, when it is symptomatic, 97% of patients present with a palpable abnormality. This is usually within a periareolar or superficial location. In a few cases, bruising and tenderness (26%), skin tethering or dimpling (14%) or nipple retraction (9%) occur. Palpable breast abnormalities that are associated with fat necrosis may enlarge, remain unchanged, regress or resolve [12, 13].

The increasing use of breast-conserving surgery and mammoplasty has given rise to a greater number of cases of fat necrosis. Middle-aged women with pendulous breasts are at the most risk [12, 13].

#### Mammography

**Fig. 5:**
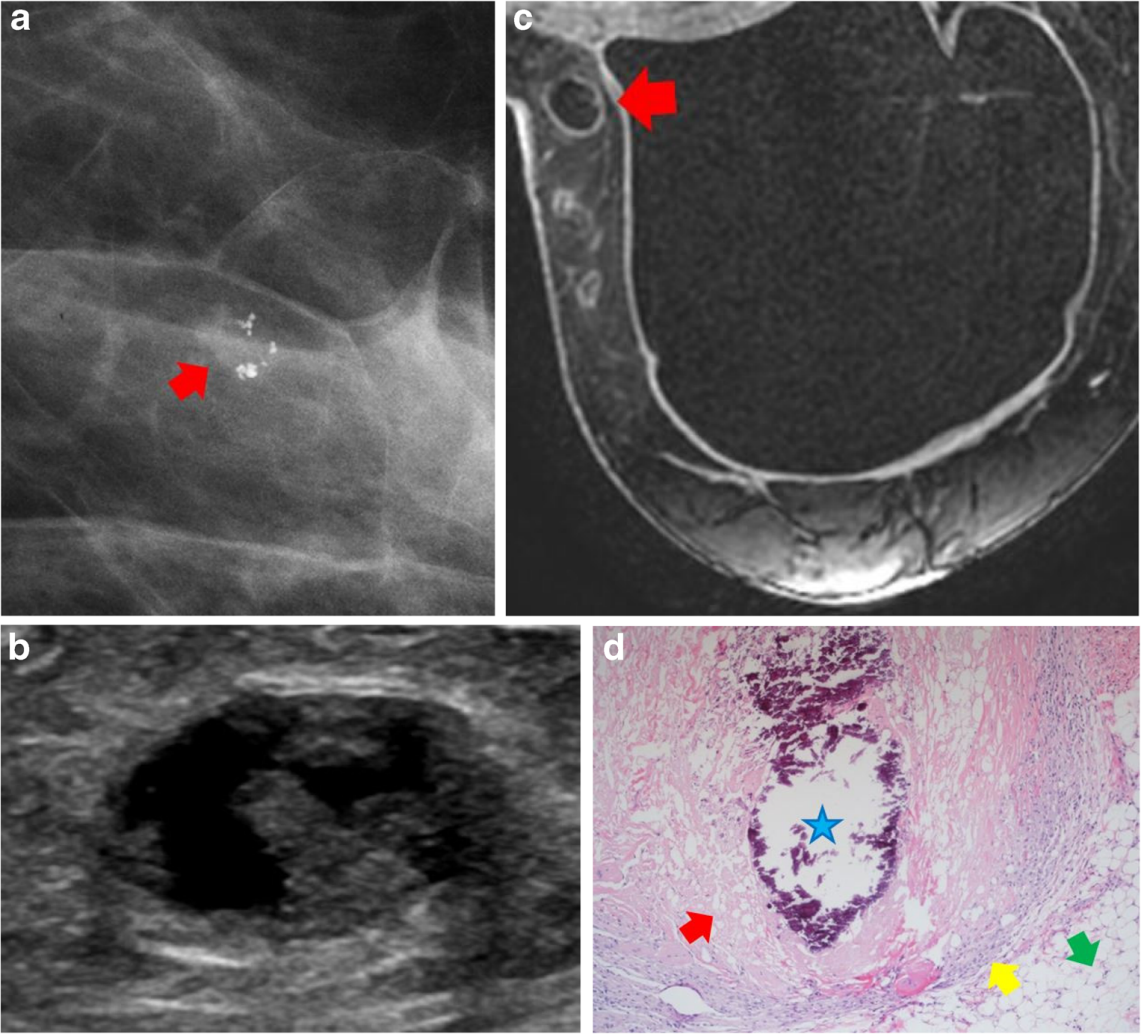
Fat necrosis. A 48-year-old woman with prior bilateral prophylactic mastectomies and implant reconstruction presented with a palpable abnormality on the inner aspect of the right breast. **a** Mammography showed new grouping of pleomorphic-appearing calcifications with associated irregular mass in the upper outer quadrant of the left breast (*red arrow*). **b** Ultrasound showed a heterogeneous, complex, cystic and solid oval mass with no vascularity. **c** Post-contrast axial MRI showed mild peripheral enhancement with central, non-enhancing fat consistent with benign fat necrosis (*red arrow*). **d** Histological photomicrograph (original magnification ×10, haematoxylin-eosin) showed calcification (*blue star*), vacuolisation and saponification of the remnants of the necrotic fat (*red arrow*), epithelioid macrophages and lymphocytes (*yellow arrow*). Normal fat is also visualised (*green arrow*)

On mammography, the most common findings of fat necrosis are oil cysts, coarse calcifications, focal asymmetries or spiculated masses, but there is a wide spectrum of mammographic imaging findings, ranging from benign to indeterminate to malignant appearing masses and calcifications. Visualised masses may be radiolucent with a thin, well-defined capsule, both radiolucent and dense with encapsulation, dense and circumscribed, or may have indistinct or spiculated margins. Lipid cysts are pathognomonic of benign fat necrosis, although the fibrous rim of the cyst may collapse and produce an appearance that is mammographically suspicious (Fig. 5).

#### Ultrasound

On sonography, the appearance of fat necrosis ranges from a solid hypoechoic mass with posterior acoustic shadowing to complex intracystic masses that evolve over time (Fig. 5). The other common sonographic finding is an area of increased echogenicity in the subcutaneous tissue with or without small cysts and architectural distortion.

#### MRI

MRI shows a wide spectrum of appearances depending on the amount of inflammatory reaction, liquefied fat and the degree of fibrosis. The necrotic central fat is isointense or slightly lower signal on saturated T1-weighted images than the remaining fat in the breast. The “Black hole” sign of fat necrosis had been described with marked hypointensity on STIR images when compared with surrounding fat. The surrounding fibrosis shows enhancement on post-gadolinium images; the enhancement being most marked in the early stages of fat necrosis and decreasing with time. The enhancement may be thin, thick, irregular or spiculated and may mimic malignancy. Kinetic analysis is of little help in granulomatous fat necrosis as the full spectrum of enhancement patterns can be seen (Fig. 5).

#### CT

CT findings of fat necrosis are not well described but tend to correspond with MRI findings with central fat and peripheral enhancement if contrast is given. The fibrosis has attenuation values similar to fibroglandular tissue or linear densities resembling fibrous bands. Calcifications occasionally are seen on CT [14].

#### Histology

Histologically, fat necrosis within the breast occurs when there is vacuolisation and saponification of the remnants of the necrotic fat, which become surrounded by lipid-laden macrophages, multinucleated giant cells and acute inflammatory cells. Fibrosis develops, enclosing the area of necrotic fat and cellular debris [12, 13, 15] (Fig. 5).

## Autoimmune causes

Although rare, granulomatous reactions to autoimmune disease have been reported in the breast including Rheumatoid disease [16], Granulomatosis with polyangiitis [17] and Sjögren’s syndrome [18].

### Sjögren’s syndrome

#### Clinical

Sjögren’s syndrome is an autoimmune disease typically affecting the salivary glands and often presents with dry eyes and mouth. Non-glandular manifestations are reported and are likely related to lymphocytic infiltration vasculitis. There is a female preponderance for Sjögren’s syndrome, and the incidence in the general population is 0.1–0.6%. There is a bimodal age distribution; peaks occur between 20 and 30 years and between 50 and 55 years. Those affected by Sjögren’s syndrome have a 5% increased risk for development of lymphoma, 18.9 times higher than that in the general population [19].

#### Mammography/US

**Fig. 6:**
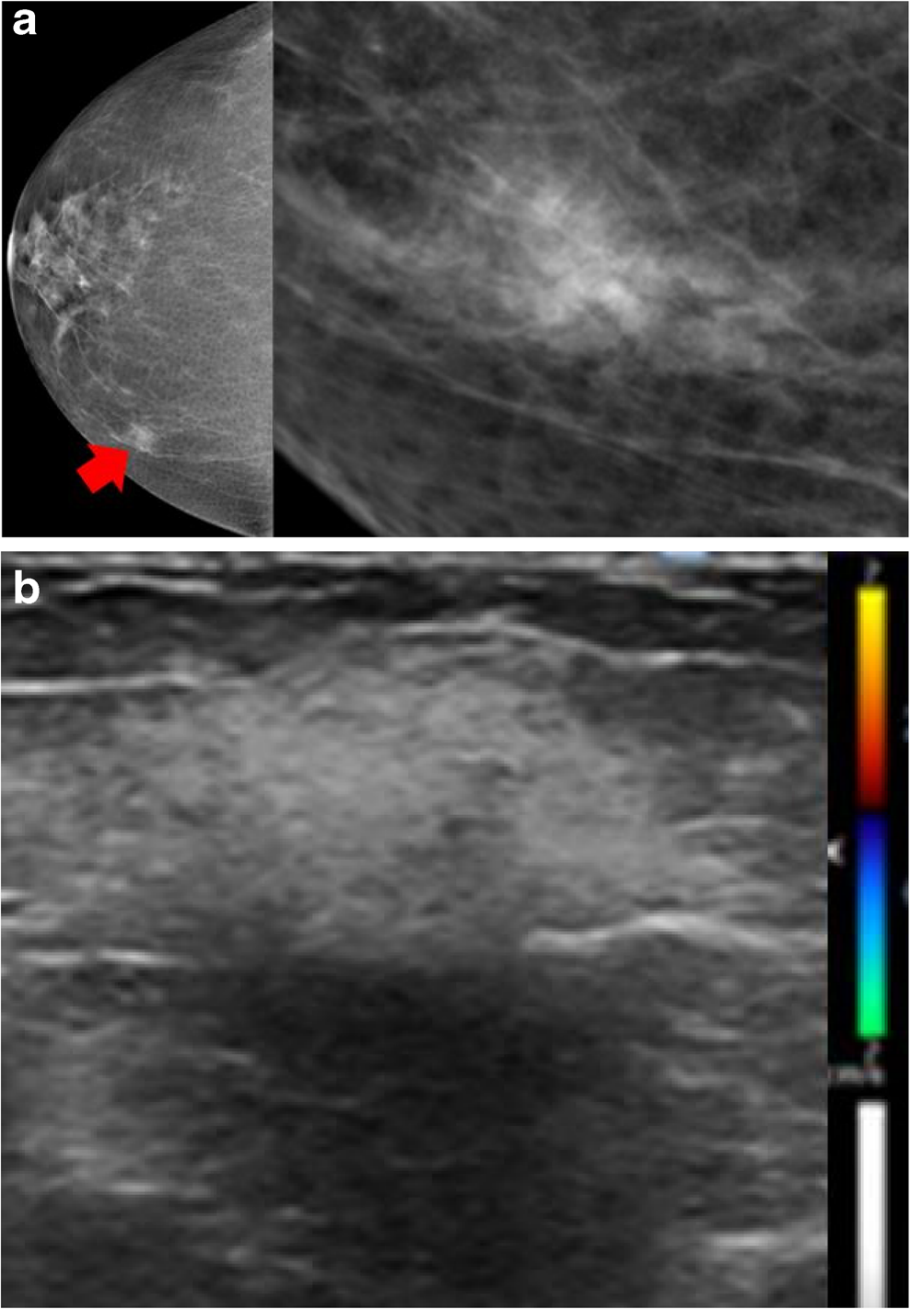
Autoimmune cause: Sjögren’s syndrome. A 61-year-old woman with Sjögren’s syndrome was positive for anti-Ro/Sjögren’s syndrome antigen A antibody. **a** Mammographic right craniocaudal with craniocaudal spot compression views showed a mass with ill-defined margins at the 2 o’clock position of the right breast (*red arrow*). **b** Ultrasound of the right breast showed an oval, hyperechoic mass with ill-defined margins, posterior shadowing and absent vascularity. Ultrasound-guided biopsy showed non-necrotising granulomatous inflammation (not shown). Fungal and acid-fast bacilli stains were negative, and angiotensin-converting enzyme and immunoglobulin G4 levels were normal. The patient received treatment for Sjögren’s syndrome, and the mass was no longer present at 6-month follow-up examination

To our knowledge, the mammographic and ultrasound findings of Sjögren’s syndrome of the breast have not been described. Our case shows a mass with ill-defined margins on mammography and an oval, hyperechoic mass with ill-defined margins, posterior shadowing, and absent vascularity on ultrasound (Fig. 6).

#### Histology

Histologically, Sjögren’s syndrome shows a chronic lymphocytic infiltration and is occasionally associated with granulomatous mastitis [18, 20].

## Granulomas of unknown cause

### Idiopathic granulomatous mastitis

As the name suggests, the cause of idiopathic granulomatous mastitis is unknown. This condition is more common in pregnant and lactating women and in patients with hyperprolactinaemia and may be related to increased ductal secretions. These increased ductal secretions may lead to damaged ductal epithelium with glandular secretions leaking into the connective tissue of the breast stroma. This process results in a chemical mastitis [21].

#### Clinical

**Fig. 7:**
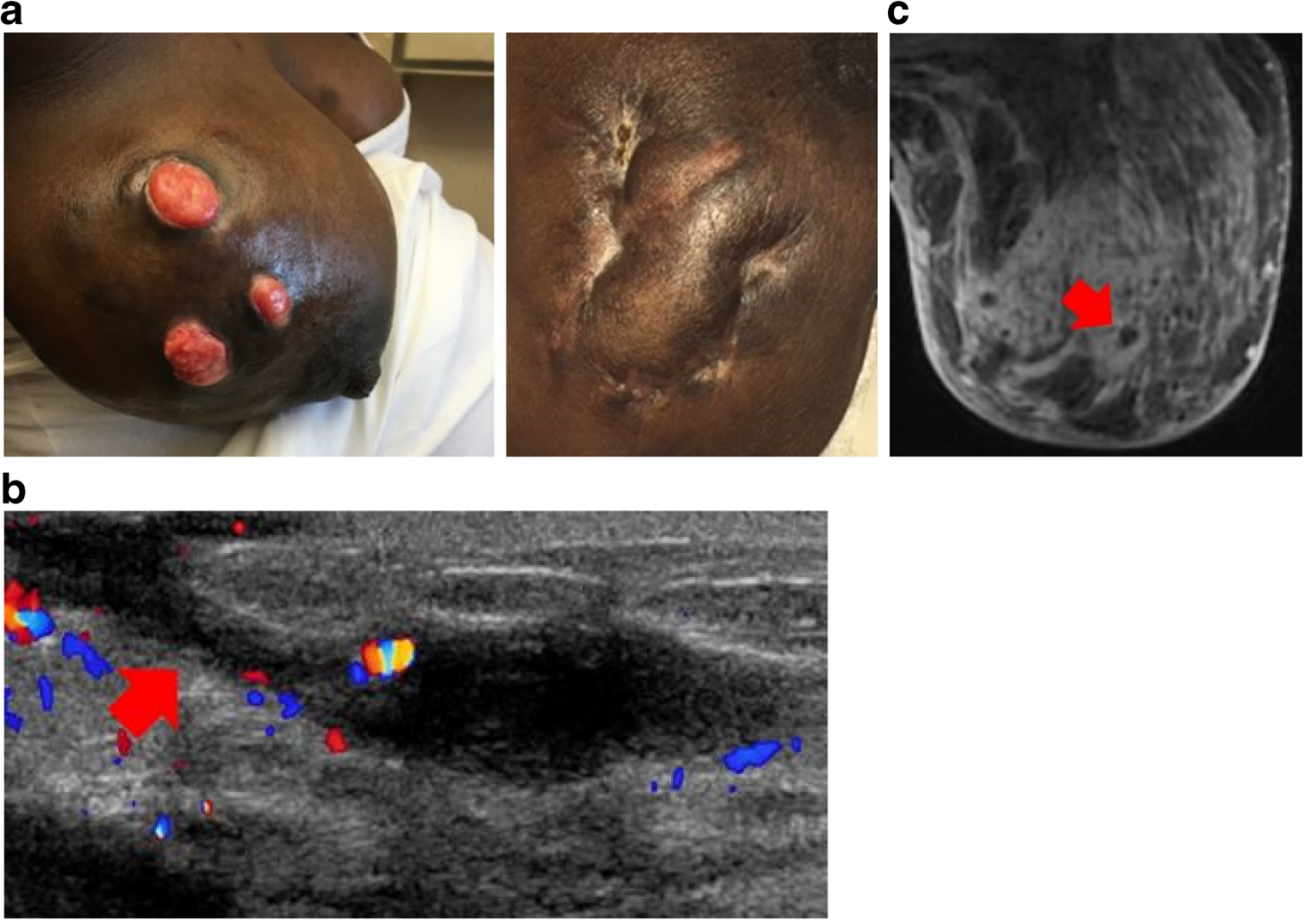
Idiopathic granulomatous mastitis. A 30-year-old woman in the second trimester of pregnancy had a tender lump in the right breast. She had first noticed a lump in the upper outer right breast approximately 2 months previously, and it had increased in size. On physical examination, there was a 10 × 10-cm firm mass in the upper outer quadrant. Biopsies showed acute and chronic granulomatous inflammation with micro-abscesses. All cultures were negative. **a** Photographs of open wounds that developed after biopsy (*left*) but healed with antibiotics but no steroids (*right*). **b** Ultrasound showed a large hypoechoic irregular mass with draining sinus tract (*red arrow*). **c** Axial post-contrast MRI showed diffuse parenchymal enhancement with micro-abscesses (*red arrow*)

Interestingly, the majority of patients are from developing countries, and further investigation into the geographic disparity may be helpful towards elucidation of a cause [22]. Idiopathic granulomatous mastitis may present in any part of the breast but tends to spare the subareolar regions. It generally manifests as a distinct, firm mass (Fig. 7). Reactive lymphadenopathy may occur in up to 15% of patients [22].

**Fig. 8:**
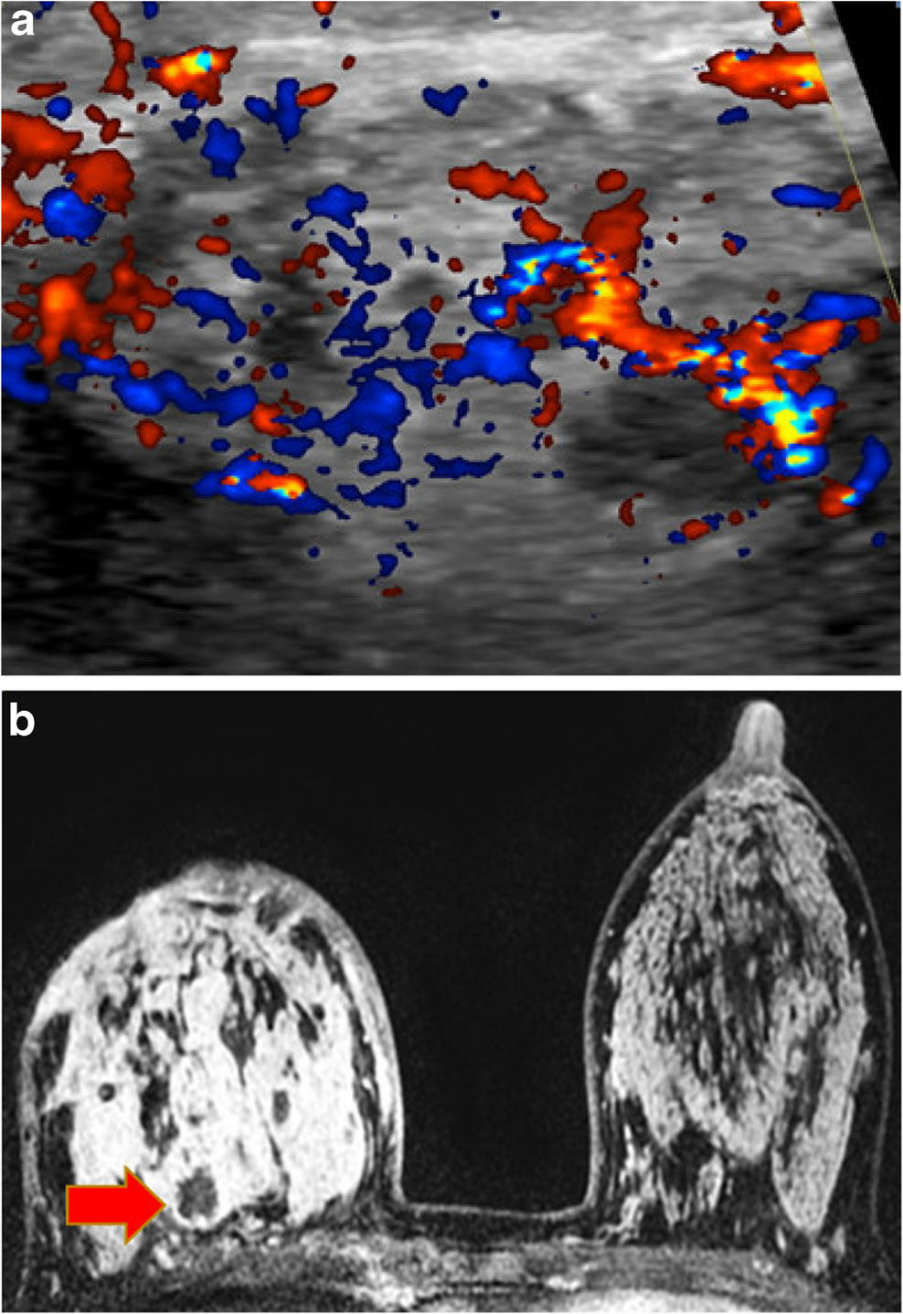
Infectious cause: *Corynebacterium*. A 31-year-old pregnant woman had a red and swollen right breast. On ultrasound-guided biopsy and aspiration, *Corynebacterium* infection was identified. **a** Ultrasound of the right periareolar region demonstrates marked vascularity on colour Doppler with heterogeneous breast tissue and areas of hypoechogenicity consistent with micro-abscesses (*red arrow*). Appearances are identical to idiopathic granulomatous mastitis sonographically. **b** Axial contrast-enhanced MRI showed global asymmetry, skin thickening, and diffuse increased enhancement in right breast with non-enhancing areas consistent with small abscess collections (*red arrow*)

Idiopathic granulomatous mastitis is frequently aggressive and demonstrates features of infectious mastitis or inflammatory carcinoma. The diagnosis is often delayed and treatment is often difficult and prolonged. The prognosis of idiopathic granulomatous mastitis is variable, and local recurrence has been reported. If the disease is localised, corticosteroid therapy has been proved effective. If it is diffuse and unresponsive to medical treatment, mastectomy may be required [22–25] (Fig. 7). Some species of *Corynebacterium* have been implicated as a cause for idiopathic granulomatous mastitis, although, strictly speaking, idiopathic granulomatous mastitis should contain no bacteria on microbiology or histology specimens [22] (Fig. 8).

#### Mammography

Mammography shows variable features, ranging from normal-appearing in patients with dense breast tissue to discrete masses. There is often focal or regional asymmetry, solitary mass or masses, skin thickening, skin or nipple retraction and axillary lymphadenopathy. The affected breast is larger than the contralateral breast and calcifications are exceedingly rare. The mammographic appearance can be indistinguishable from invasive or inflammatory breast cancer.

#### Ultrasound

On ultrasonography, multiple contiguous hypoechoic masses have been considered suggestive of the disease with skin fistulae common in advanced cases. Posterior acoustic enhancement and shadowing have both been described. Doppler imaging demonstrates increased vascularity and in advanced cases fluid collections and cavities can be seen. Ultrasound is useful in documenting sinus tracts and in the evaluation of any enlarged axillary reactive lymph nodes.

#### MRI

MRI is indicated to assess the extent of disease and in assessment of the contralateral breast. It is best reserved for advanced, aggressive or refractory disease and can be used to assess disease progression or regression over time. The MRI findings are variable depending on the severity of the inflammation, with both heterogeneous ill-defined masses and non-mass enhancement being described [22]. Idiopathic granulomatous mastitis demonstrates marked parenchymal enhancement most often with progressive or plateau kinetics. There are often areas of increase T2 signal representing fluid collections. The involved parenchyma demonstrates restricted diffusion with ADC valves consistently lower than seen in normal breast parenchyma [22].

#### Histology

On histopathologic examination, non-caseating granulomatous inflammation is centred on lobules. Common end-stage features of idiopathic granulomatous mastitis are fat necrosis, abscess formation and fibrosis [22, 26, 27].

### Sarcoidosis

The aetiology of sarcoidosis is unknown, and usually the lungs, lymph nodes, skin, spleen, and liver are involved. Breast involvement is rare, occurring in less than 1% of patients with systemic sarcoidosis.

#### Clinical

Mammary lesions are usually detected after the diagnosis of sarcoidosis has already been well established, and only rarely does sarcoidosis present initially as a breast disease. Like sarcoidosis elsewhere, it usually affects women in their 20s and 30s and can manifest as a firm, hard mass mimicking breast cancer or lymphadenopathy. In 20% of reported cases of sarcoidosis of the breast, the initial presentation is a breast mass. The characteristic clinical finding is a non-tender, mobile breast mass with other organ involvement and an increased level of angiotensin-converting enzyme [3–5, 28].

#### Mammography

**Fig. 9:**
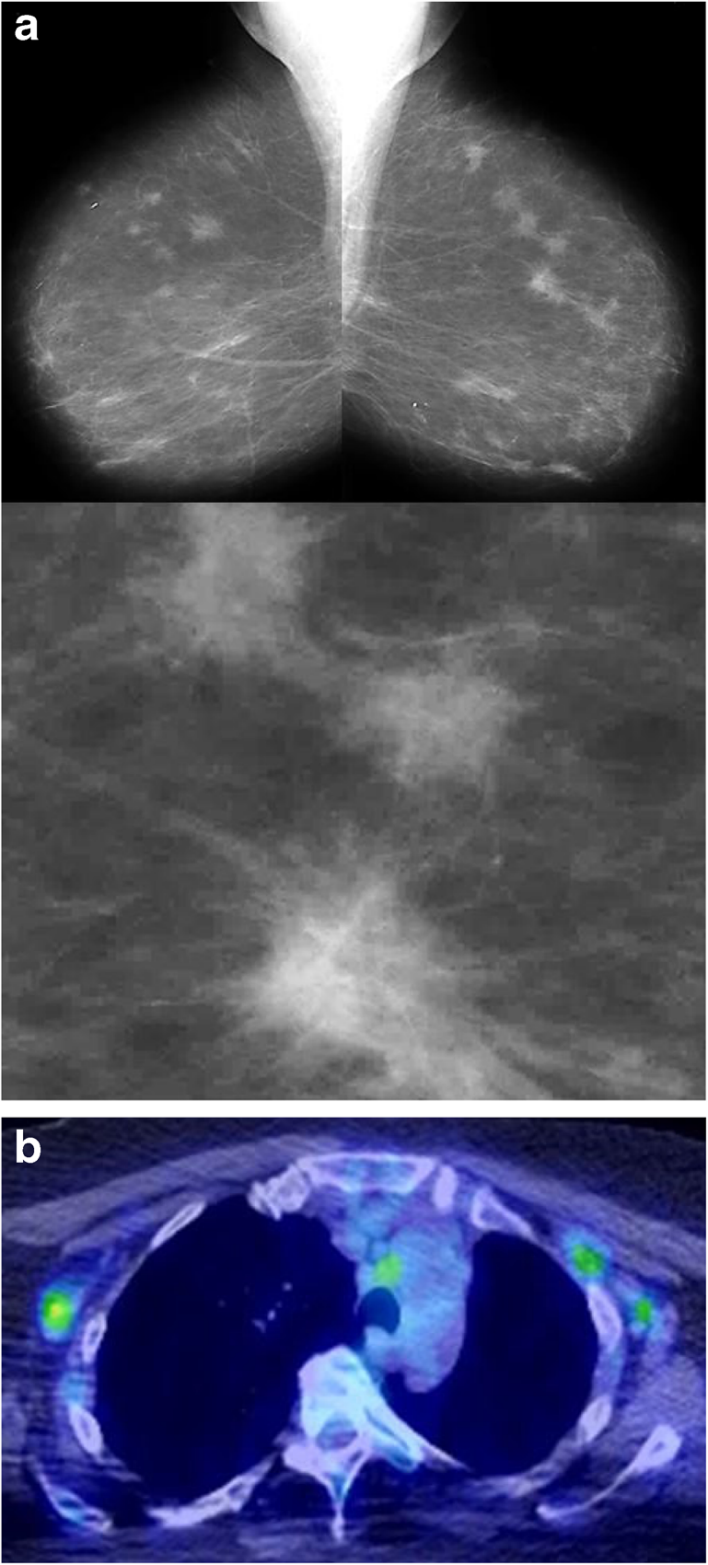
Sarcoidosis. A 72-year-old woman had a prior skin biopsy showing sarcoidosis. **a** Bilateral mediolateral-oblique views and magnification view show multiple spiculated masses in both breasts. These had been unchanged for 5 years. **b** Positron emission tomography showed multiple areas of increased uptake in mediastinal and axillary lymph nodes. Histopathological examination of left breast ultrasound-guided biopsy specimens showed non-caseating granulomas consistent with sarcoidosis (not shown)

On mammography, breast sarcoidosis can present as bilateral, irregular or spiculated masses (Fig. 9). Very small, well-defined round masses have also been described and may represent intramammary lymph node involvement. Calcifications are typically not seen.

#### Ultrasound

Irregular, hypoechoic masses with indistinct margins are the most common finding on ultrasonography.

#### MRI

Various appearances have also been observed. Inhomogeneous signal intensity masses with irregular contours, rapid enhancement and early “washout” are most common [5, 6, 28].

#### PET/CT

Increased uptake on positron emission tomography can cause sarcoidosis to be mistaken for metastatic breast carcinoma (Fig. 9). It is important for clinicians and radiologists to be aware that imaging findings and systemic manifestations of sarcoidosis can be confused with those of metastatic breast cancer [29, 30].

#### Histology

Gross pathological findings consist of firm to hard, tan tissue that may have well-defined or indistinct borders and can measure up to 5 cm in diameter. Microscopic examination shows epithelioid granulomas with multinucleated giant cells. The lesions classically do not have caseous necrosis or calcification, and fat necrosis is not found in the surrounding breast. Associated lymphocytes and fibrosis are often present. Not all non-necrotising granulomas are sarcoidosis; however, other causes of non-necrotising granulomas need to be excluded. The diagnosis requires exclusion of these other causes and often clinical evidence of sarcoidosis elsewhere. Stains and cultures for bacteria, mycobacteria and fungus should be negative. These studies are indicated even in patients with previously diagnosed sarcoidosis, because secondary infections can develop, especially in patients who have had corticosteroid treatment.

## Axillary lymph nodes

### Granulomatous lymphadenitis

The causes of granulomas within axillary lymph nodes are similar to the causes of granulomas within the breast parenchyma. Lymph node granulomas are often classified into infectious or non-infectious causes.

Non-infectious causes of granulomatous lymphadenitis include sarcoidosis, berylliosis and sarcoid-like reaction, and these are rarely associated with abscesses or necrosis [31]. Infectious granulomatous lymphadenitis can be further categorised as suppurative or non-suppurative. Suppurative infections are associated with central abscesses, necrosis and granulomas and include tularaemia, cat-scratch disease, yersiniosis and lymphogranuloma venereum.

#### Clinical

Patients present with tenderness, erythema, swelling and abscess formation. Alternatively, non-suppurative infectious granulomatous lymphadenitis shows no signs of inflammation and results in “cold” abscesses. Causes of non-suppurative infections include tuberculous, atypical mycobacterium, toxoplasmosis, syphilis, brucellosis or various fungal infections [31].

#### Mammography/ultrasound

**Fig. 10:**
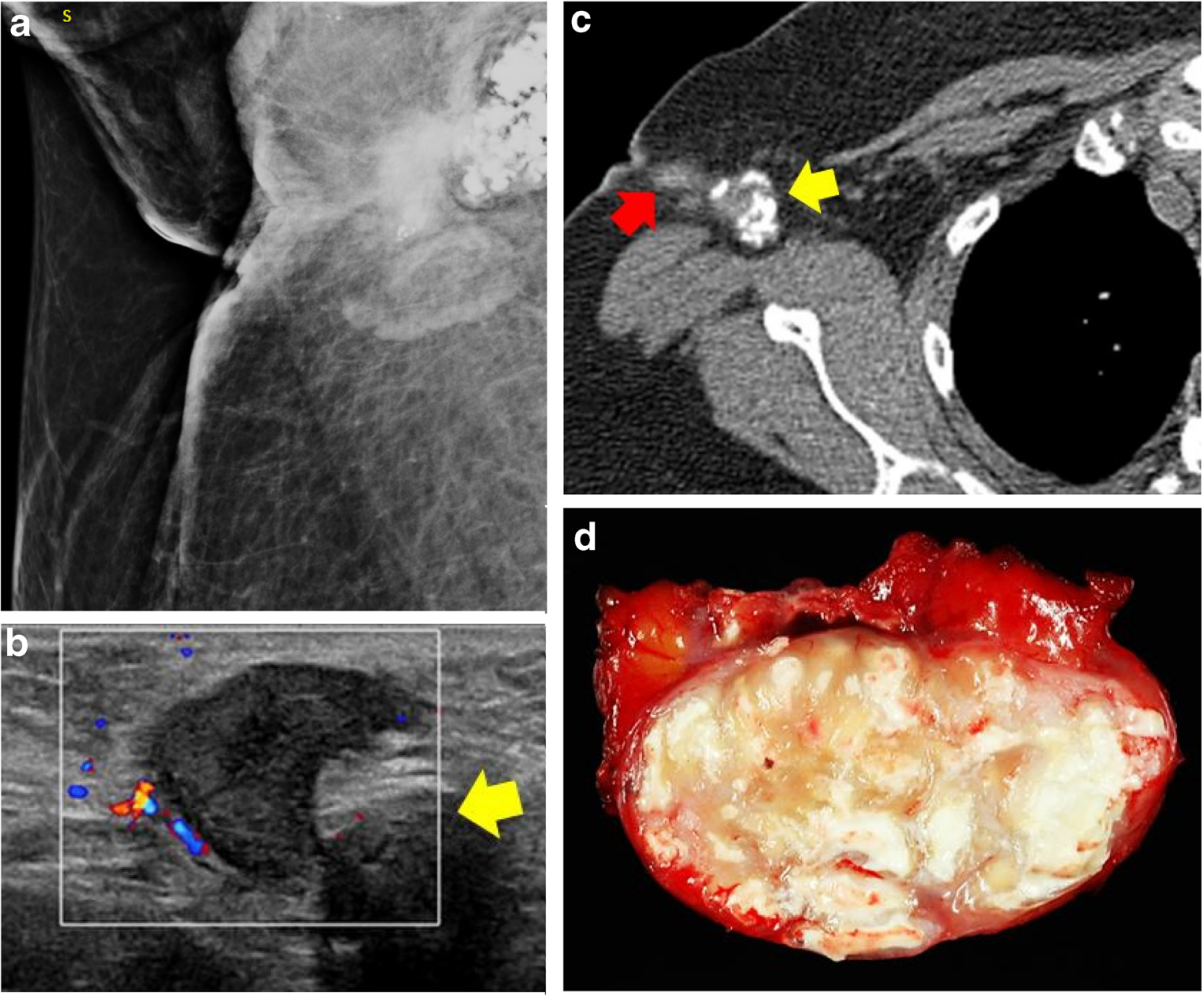
Granulomatous lymphadenitis. A 44-year-old woman had a tender lump in the right axillary region with recurrent draining sinuses. **a** Mammography showed a predominantly calcified right axillary mass with irregular mass and associated skin thickening. **b** Ultrasound showed multiple hypoechoic round and irregular masses in right axilla, some with calcifications (*yellow arrow*). **c** CT showed the calcified right axillary lymph node (*yellow arrow*) and sinus tract (*red arrow*). **d** Gross pathological specimen showed organising abscess with acute inflammation and fat necrosis. Special stains for bacteria, fungi and mycobacteria were negative

On imaging, the findings of granulomatous lymphadenitis are non-specific and can mimic those of metastatic adenopathy. Mammographically and sonographically, lymph nodes may appear enlarged, round and of high density, and are commonly thickened with loss of their fatty hilum [31]. Occasionally these lymph nodes calcify (Fig. 10).

### Cat-scratch disease

Although granulomatous lymphadenitis of the axilla has multiple causes, only a few specifically have a preference for the axilla and typically spare the breast parenchyma. Cat-scratch disease, or cat-scratch fever, is one of those processes. It is caused by a bacterial infection and most commonly occurs in children after a cat scratches or bites an arm [12]. It has worldwide distribution and has been described in all areas of North America. In northern temperate zones, it occurs more often from August through October, usually in humid, warm locales. There are an estimated 22,000 new cases of cat-scratch disease per year in the United States [32].

#### Clinical

Cat-scratch disease is usually limited to one side of the body and commonly presents as tender, swollen lymph nodes near the site of the bite or scratch. Regional lymphadenopathy occurs 1–3 weeks after inoculation. A vesicle or an erythematous papule may form at the site of initial infection, and regional lymphadenopathy (85%), axillary lymphadenopathy (27%), and fever, headache and malaise (77%) often develop. It may take 7–14 days before symptoms appear, and most cases are self-limited, resolving within 1 month with or without treatment. The lymphadenopathy may, however, persist for several months after the other symptoms have disappeared [33].

#### Mammography/ultrasound

**Fig. 11:**
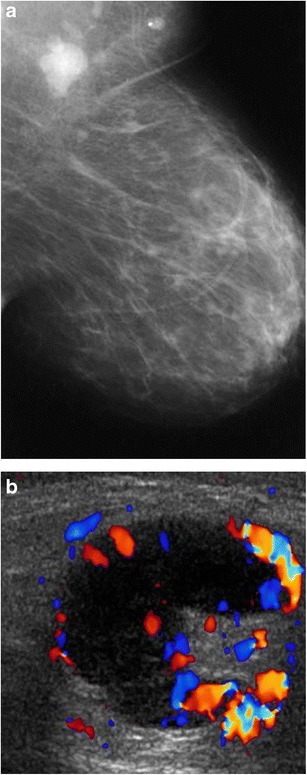
Cat-scratch disease (*Bartonella henselae*). A 59-year-old woman had a 1-month history of a left axillary mass. Ultrasound-guided biopsy showed follicular hyperplasia with non-necrotising epithelioid granulomas. *B. henselae* (cat-scratch disease) titre was more than 1:1,024, consistent with recent infection. **a** Left mediolateral-oblique view with magnified image showed a new left axillary mass (*arrow*). **b** Ultrasound showed multiple enlarged lymph nodes in the left axilla with lobulated margins, thickened cortices and increased cortical vascular flow

Lymph nodes are enlarged on mammography. On ultrasound they are hypoechoic lobular or oval masses with central hyperaemia and occasional adjacent fluid. The presence of asymmetry and a hyperechoic hilum may differentiate cat-scratch disease from other aetiologies (Fig. 11).

#### Histology

Cat-scratch disease is characterised by granulomatous inflammation on histological examination of the lymph nodes. Breast parenchymal involvement occurs less commonly; when it does, it presents as a palpable mass with enlarged lymph nodes within the axilla in 38% of patients [33].

## Conclusions

Granulomatous processes of the breast and axilla are rare with variable imaging findings that can mimic malignancy. The diagnosis can, however, be made if there is relevant history (prior trauma, silicone breast implants, lactation), laboratory (systemic or infectious processes) and imaging findings (fistula, nipple retraction) (Table 1).

**Table 1:** Clinical and imaging findings of granulomatous processes of the breast and axilla

	Clinical	Imaging
Infectious	• Aspiration demonstrating bacterial, fungal or parasitic infection	• Mammo: serpiginous calcifications seen with parasitic infection • US/MRI: lymphadenopathy, skin thickening, ill-defined mass, often with sinus tracts and fistulae
Fat necrosis	• History of trauma	• Mammo: lipid cysts or dystrophic calcifications • US/MRI/CT: fat seen within the centre of mass
Suture	• Prior surgery	• Mammo: Suture knots seen on mammography
Silicone	• Breast implants	• Mammo: high-density silicone on mammography • US: snow storm on US • MRI: high-signal on silicone-sensitive sequences • CT: dual-energy CT identifying silicone
Autoimmune	• Rheumatoid: cutaneous rheumatoid nodules, RF, ANA • GPA: ANCA • Sjögren’s syndrome: dry eyes and mouth, Anti-Ro/SSA, Anti-La/SSB	• Mammo: bilateral ill-defined or irregular masses • US: Bilateral irregular hypoechoic masses
Sarcoidosis	• ACE elevated, hypercalcaemia or hypercalciuria • Lungs, skin or lymph nodes, less common eyes, liver, heart & brain	• Mammo/US: bilateral masses, asymmetry or architectural distortion • [^18^F]-FDG-PET: Increased uptake
IGM	• Pregnant or lactating • Subareolar or entire breast • Affected breast larger, with pain, erythema, swelling or axillary lymphadenopathy • Cultures negative	• Mammo/US: solitary mass, skin thickening and nipple retraction • MRI: marked parenchymal enhancement with sterile micro-abscesses
Lymph nodes	• Similar aetiologies to breast • Infectious vs non-infectious • Cat bite or scratch to arm	• Mammo/US: lymph nodes enlarged and round • Fistula to skin

*ANA* antinuclear antibody, *ANCA* antineutrophil cytoplasmic antibody, *ACE* angiotensin-converting enzyme, *FDG-PET* fluorodeoxyglucose positron emission tomography, *GPA* granulomatosis with polyangiitis, *IGM* idiopathic granulomatous mastitis, *Mammo* mammography, *RF* rheumatoid factor, *US* ultrasound

Granulomatous breast disease commonly presents with a unilateral breast mass, and axillary lymphadenopathy can be seen. Fistula formation can suggest a granulomatous cause—either infectious or idiopathic granulomatous mastitis. Mammographic findings include asymmetric density or an ill-defined mass. Sonographic findings commonly demonstrate an irregular hypoechoic mass or masses with specific findings for granulomas, including: the “snowstorm” appearance seen with extracapsular silicone; ill-defined confluent hypoechoic areas with marked vascularity and sinus tracts to the skin, as well as adjacent fluid collections, seen with *Mycobacterium* infections and idiopathic granulomatous mastitis; circumscribed hypoechoic masses with or without surrounding hyperechogenicity, which can be found with fat necrosis. MRI is beneficial in evaluation of the extent of disease and may demonstrate peripheral enhancement and abscess formation as the granuloma tries to wall-off the inciting antigen.

Recognising these entities is important for establishing pathological concordance after biopsy and for preventing unnecessary treatment.

## References

[1] Schnarkowski P, Schmidt D, Kessler M, Reiser MF (1994). Tuberculosis of the breast: US, mammographic, and CT findings. J Comput Assist Tomogr.

[2] Oh KK, Kim JH, Kook SH (1998). Imaging of tuberculous disease involving breast. Eur Radiol.

[3] Fitzgibbons PL, Smiley DF, Kern WH (1985). Sarcoidosis presenting initially as breast mass: report of two cases. Hum Pathol.

[4] Banik S, Bishop PW, Ormerod LP, O’Brien TE (1986). Sarcoidosis of the breast. J Clin Pathol.

[5] Ishimaru K, Isomoto I, Okimoto T, Itoyanagi A, Uetani M (2002). Sarcoidosis of the breast. Eur Radiol.

[6] Kirshy D, Gluck B, Brancaccio W (1999). Sarcoidosis of the breast presenting as a spiculated lesion. AJR Am J Roentgenol.

[7] Robinson OG, Bradley EL, Wilson DS (1995). Analysis of explanted silicone implants: a report of 300 patients. Ann Plast Surg.

[8] Silverman BG, Brown SL, Bright RA, Kaczmarek RG, Arrowsmith-Lowe JB, Kessler DA (1996). Reported complications of silicone gel breast implants: an epidemiologic review. Ann Intern Med.

[9] Kasper CS (1994). Histologic features of breast capsules reflect surface configuration and composition of silicone bag implants. Am J Clin Pathol.

[10] Council on Scientific Affairs, American Medical Association (1993). Silicone gel breast implants. JAMA.

[11] Glazebrook KN, Leng S, Jacobson SR, McCollough CM (2016) Dual-energy CT for evaluation of intra- and ectracapsular silicone implant rupture. Case Rep Radiol 2016:6323709 10.1155/2016/6323709 PMC4749786 26942031

[12] Taboada JL, Stephens TW, Krishnamurthy S, Brandt KR, Whitman GJ (2009). The many faces of fat necrosis in the breast. AJR Am J Roentgenol.

[13] Hogge JP, Robinson RE, Magnant CM, Zuurbier RA (1995). The mammographic spectrum of fat necrosis of the breast. Radiographics.

[14] Chala LF, de Barros N, de Camargo MP, Endo E, Kim SJ, Pincerato KM (2004). Fat necrosis of the breast: mammographic, sonographic, computed tomography, and magnetic resonance imaging findings. Curr Probl Diagn Radiol.

[15] Kerridge WD, Kryvenko ON, Thompson A, Shah BA (2015). Fat necrosis of the breast: a pictorial review of the mammographic, ultrasound, CT, and MRI findings with histopathologic correlation. Radiol Res Pract.

[16] Cooper NE (1991). Rheumatoid nodule in the breast. Histopathology.

[17] Deininger HK (1985). Wegener granulomatosis of the breast. Radiology.

[18] Letourneux C, Diemunsch P, Korganow AS, Akladios CY, Bellocq JP, Mathelin C (2013). First report of granulomatous mastitis associated with Sjögren’s syndrome. World J Surg Oncol.

[19] Miller AV, Ranatunga SK, Tumyan A, Francis ML, Pema K. Sjögren syndrome. Available from: http://emedicine.medscape.com/article/332125-overview#a7

[20] Sumikawa Y, Ansai S, Kimura T, Nakamura J, Inui S, Katayama I (2010). Interstitial type granuloma annulare associated with Sjögren’s syndrome. J Dermatol.

[21] Erhan Y, Veral A, Kara E (2000). A clinicopthologic study of a rare clinical entity mimicking breast carcinoma: idiopathic granulomatous mastitis. Breast.

[22] Fazzio RT, Shah SS, Sandhu NP, Glazebrook KN (2016). Idiopathic granulomatous mastitis: imaging update and review. Insights Imaging.

[23] Memis A, Bilgen I, Ustun EE, Ozdemir N, Erhan Y, Kapkac M (2002). Granulomatous mastitis: imaging findings with histopathologic correlation. Clin Radiol.

[24] Yilmaz E, Lebe B, Usal C, Balci P (2001). Mammographic and sonographic findings in the diagnosis of idiopathic granulomatous mastitis. Eur Radiol.

[25] Han BK, Choe YH, Park JM (1999). Granulomatous mastitis: mammographic and sonographic appearances. AJR Am J Roentgenol.

[26] Fletcher A, Magrath IM, Riddell RH, Talbot IC (1982). Granulomatous mastitis: a report of seven cases. J Clin Pathol.

[27] Going JJ, Anderson TJ, Wilkinson S, Chetty U (1987). Granulomatous lobular mastitis. J Clin Pathol.

[28] Kenzel PP, Hadijuana J, Hosten N, Minguillon C, Oellinger H, Siewert C (1997). Boeck sarcoidosis of the breast: mammographic, ultrasound, and MR findings. J Comput Assist Tomogr.

[29] Ito T, Okada T, Murayama K (2010). Two cases of sarcoidosis discovered accidentally by positron emission tomography in patients with breast cancer. Breast J.

[30] Bush E, Lamonica D, O’Connor T (2011). Sarcoidosis mimicking metastatic breast cancer. Breast J.

[31] Asano S (2012). Granulomatous lymphadenitis. J Clin Exp Hematop.

[32] Jackson LA, Perkins BA, Wenger JD (1993). Cat scratch disease in the United States: an analysis of three national databases. Am J Public Health.

[33] Klotz SA, Ianas V, Elliott SP (2011). Cat-scratch disease. Am Fam Physician.

